# Supplementary material for: Chemokine induces phase transition from non-directional to directional migration during angiogenesis
Source: Cell Struct Funct. 2025 Mar 13;50(1):91–101. doi: 10.1247/csf.24081 (PMC12702683; doi:10.1247/csf.24081)
Supplement: Supplementary file 5 — Supplementary Materials [file csf_50_24081_5.zip › 50_24081_Supple.docx]

**Supplementary Materials**

**Supplementary Figures**

**
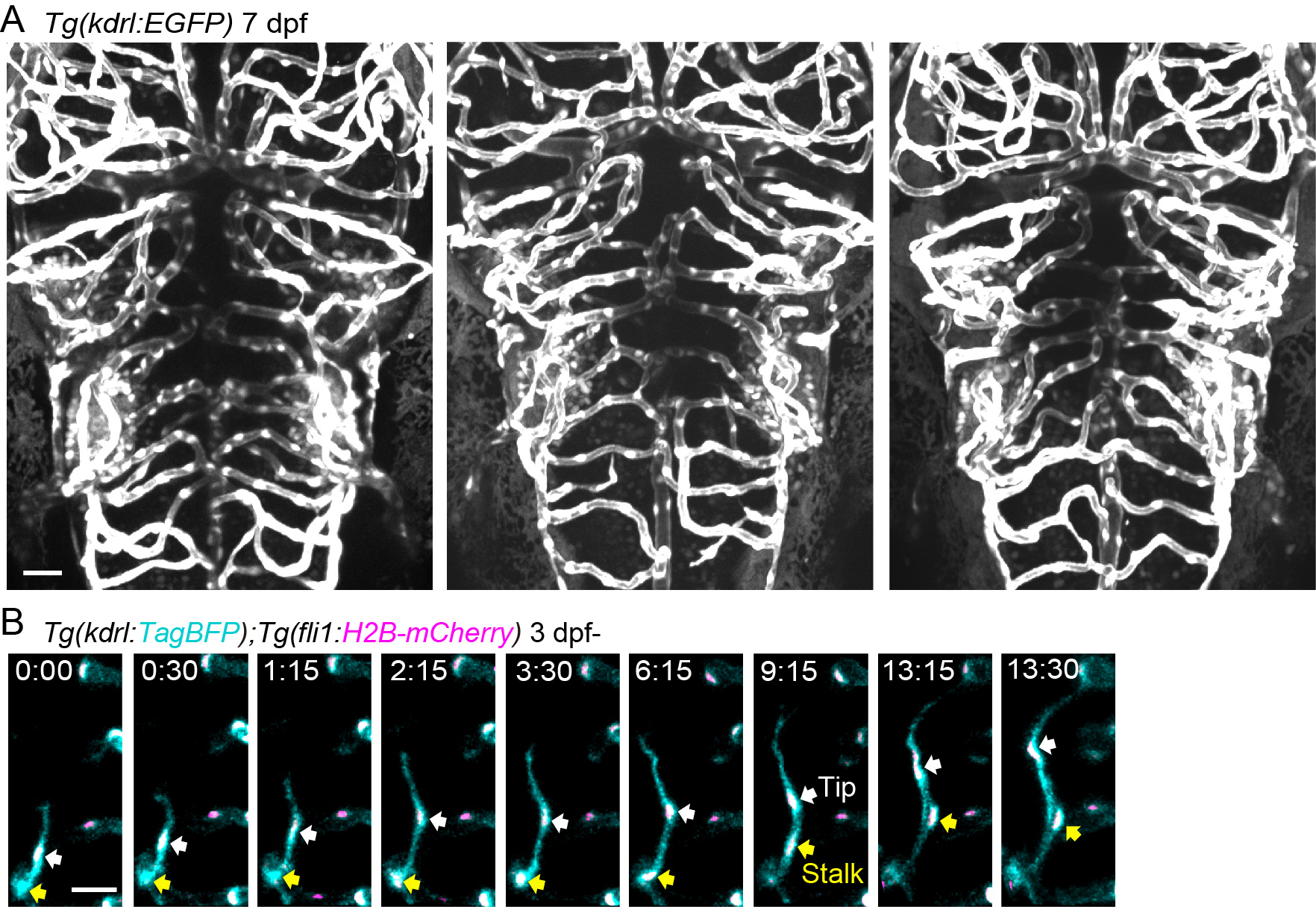
**

**Figure S1 Formation of capillary networks in the zebrafish brain.**

(A) Representative confocal images of the brain of *Tg(kdrl:EGFP-CAAX)* larvae (7 dpf). *kdrl*:EGFP^+^ endothelial cells (ECs) are shown as white. Dorsal view, anterior to the top. Central artery (CtA) networks show different patterns in different individuals.

(B) Time-sequential images of a *Tg(kdrl:TagBFP)*;*Tg(fli1:H2B-mCherry)* larva (from 3 dpf). Elapsed time (h:min). Tip cell nucleus and stalk cell nucleus are pointed by white arrowheads and yellow arrowheads, respectively. In this sprout, directional migration of the tip cell transiently stopped (2:15 to 9:15) and restarted after the stalk cell (yellow arrowheads) migrated out of the parental vessel.

Scale bar:10 μm.

**
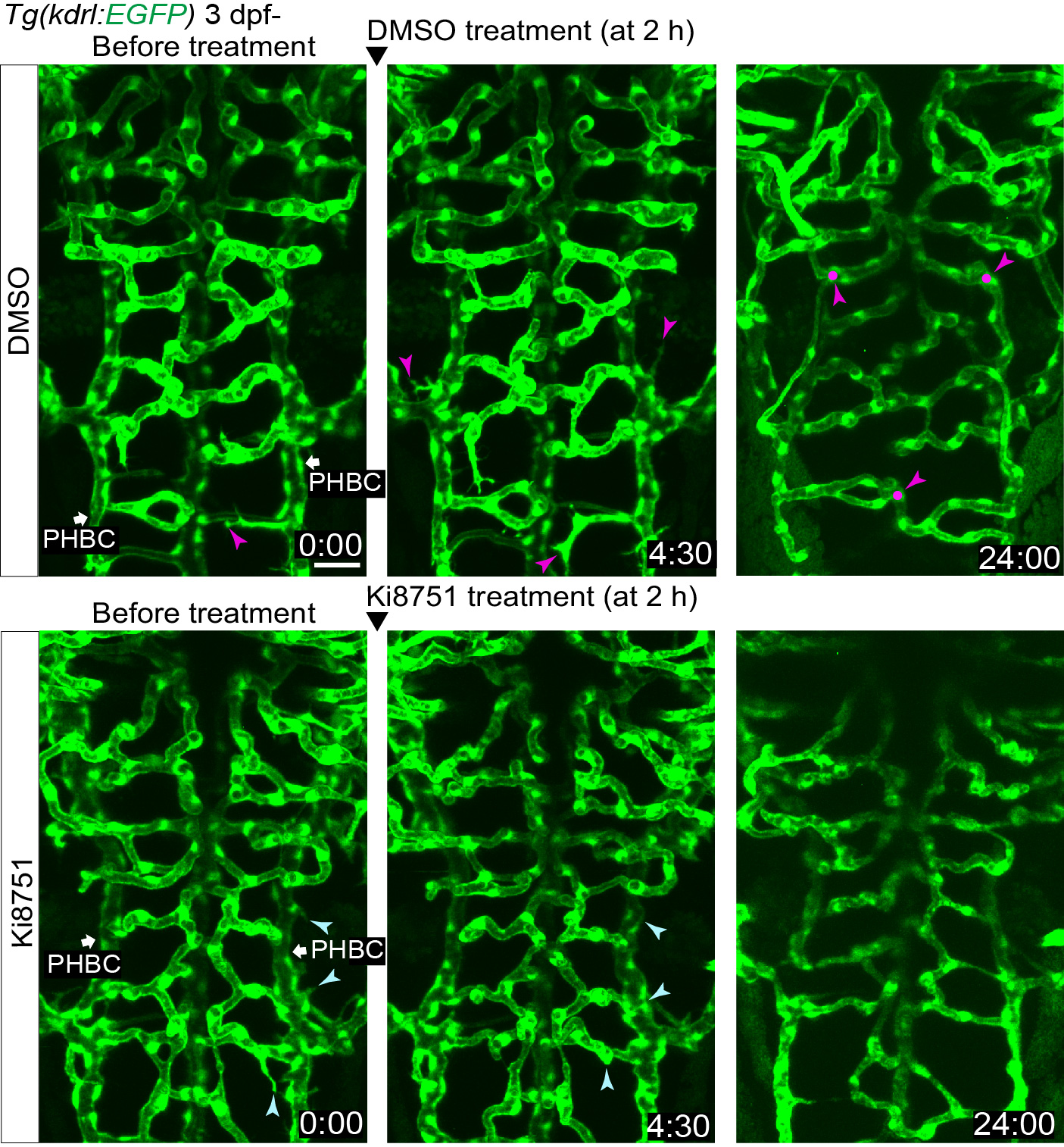
**

**Figure S2 Inhibition of tip cell sprouting in Vegfr2-inhibited larvae.**

Time-sequential images of *Tg(kdrl:EGFP)* larvae (from 3 dpf) treated with DMSO or 1 μM Ki8751 at 2 h after the start of imaging. Elapsed time (h:min). ECs sprout from the primordial hindbrain channels (PHBC) (magenta arrowheads) and connect with the CtAs (magenta circles) in DMSO-treated larvae, whereas ECs never sprout after Ki8751 treatment. ECs that had already sprouted are retracted soon after Ki8751 treatment (blue arrowheads).

Scale bar:10 μm. PHBC, primordial hindbrain channel.

**
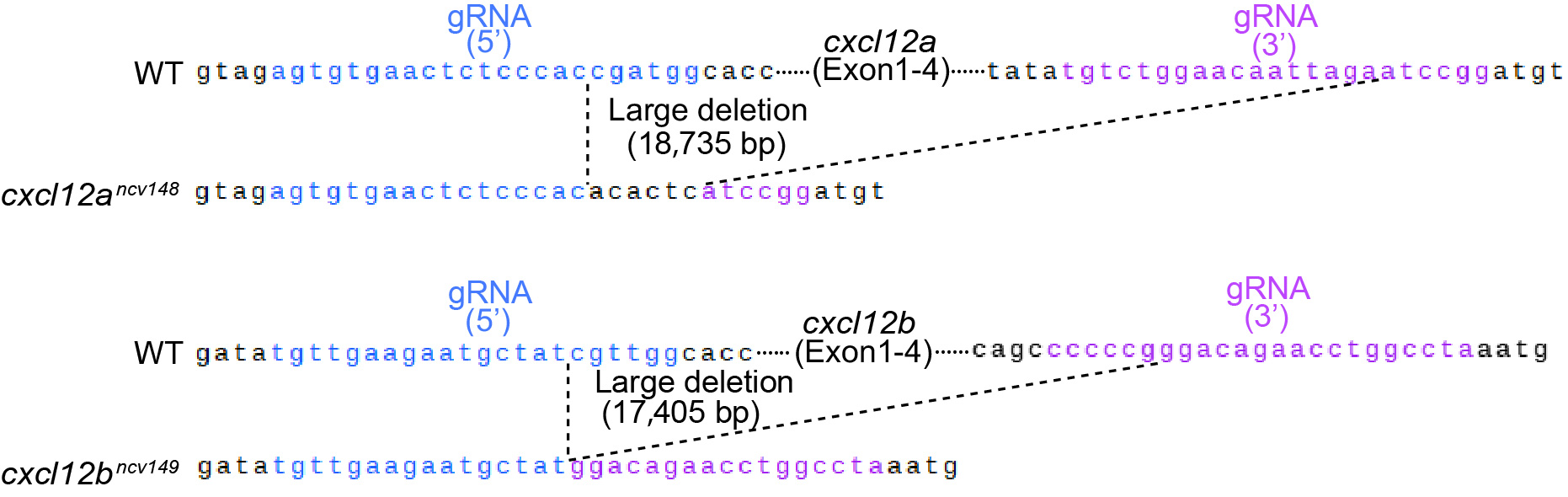
**

**Figure S3 DNA sequence of full locus deletion fish of *cxcl12a* and *cxcl12b*.**

DNA sequence of zebrafish *cxcl12a* and *cxcl12b* in WT and mutants. The *cxcl12a^ncv148^* and *cxcl12b^ncv149^* alleles harbor 18,735 base pair (bp) and 17,405 bp deletions, respectively, leading to the loss of all exons. In both genes, double guide RNAs (gRNAs) were designed upstream of the 5' UTR (blue) and downstream of the 3' UTR (purple), flanking all exons.

**
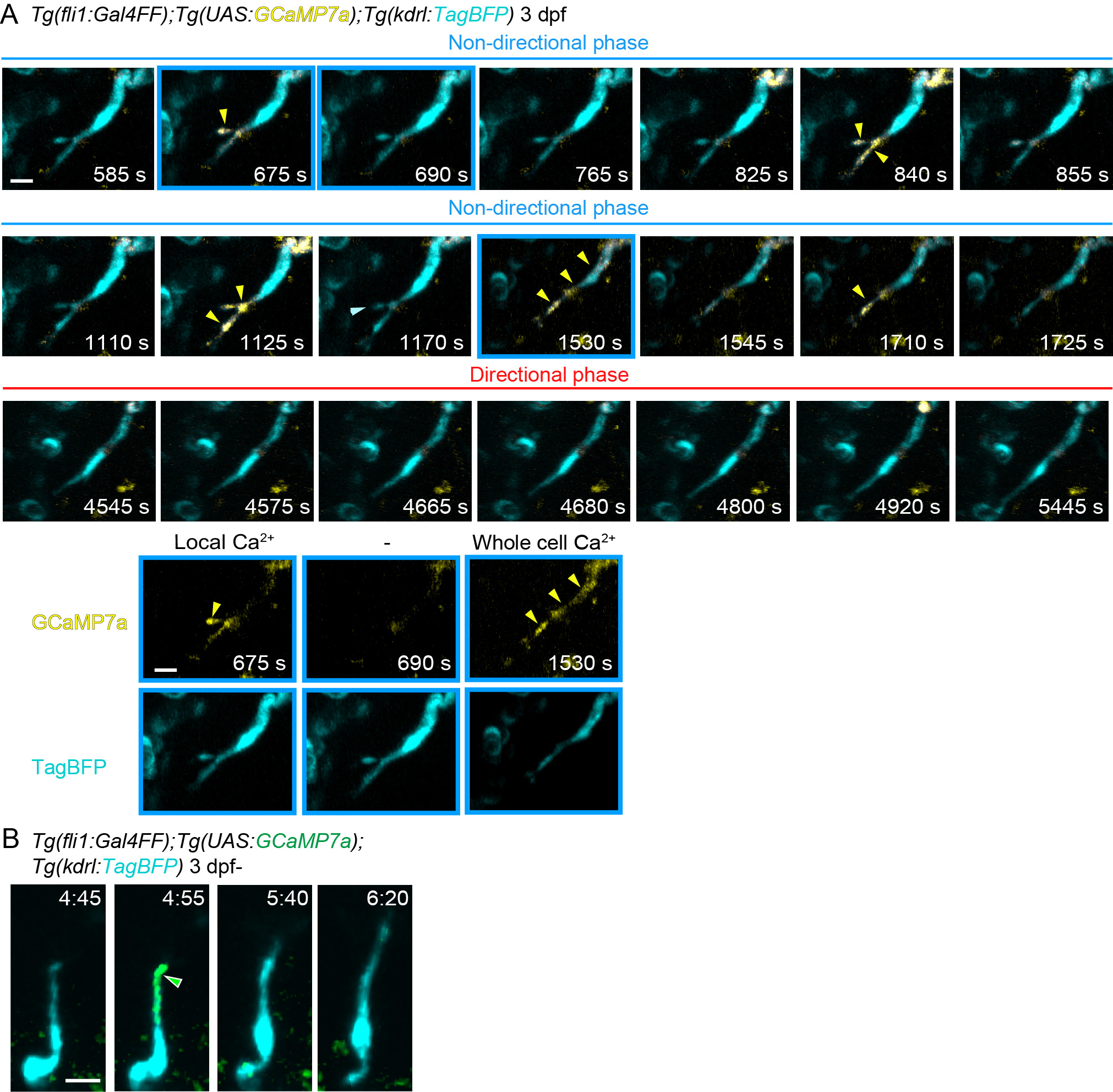
**

**Figure S4 Ca^2+^ dynamics in tip cells sprouting from the PHBC.**

(A) Time-sequential images of high-speed 3D time-lapse imaging of a *Tg(fli1:Gal4FF);Tg(UAS:GCaMP7a);Tg(kdrl:TagBFP)* larva (3 dpf) taken every 15 sec. In addition to the whole cell Ca^2+^ increase (for example, at 1530 s), local Ca^2+^ oscillations are detected at the leading front of a tip cell in the non-directional phase (yellow arrowheads), but rarely in the directional phase. Elapsed time (sec (s)). The images at 675 s, 690 s, and 1530 s are described in single color as representative of local Ca^2+^ increase, no Ca^2+^ increase, and the whole cell Ca^2+^ increase, respectively (lower panels).

(B) Time-sequential images of a *Tg(fli1:Gal4FF);Tg(UAS:GCaMP7a);Tg(kdrl:TagBFP)* larva (from 3 dpf) after tip cell budding from the PHBC. Local Ca^2+^ increases in an extending protrusion in the non-directional phase (green arrowhead).

Scale bar:10 μm.

**
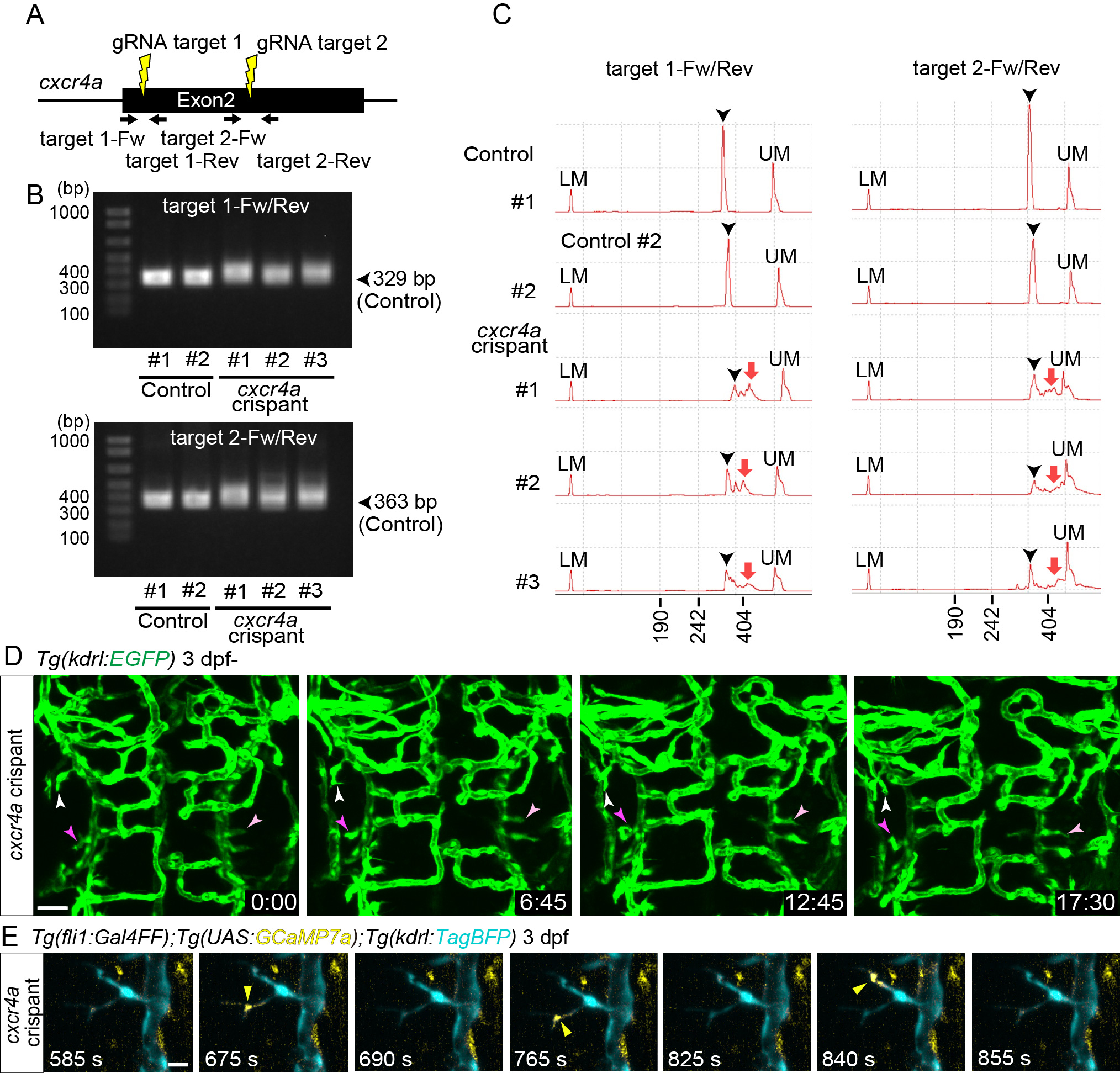
**

**Figure S5 Phenotypes of *cxcr4a* crispants in cerebral vascular development.**

(A) Schematic representation for generating *cxcr4a* F0 knockout zebrafish using double *cxcr4a* gRNAs (gRNA target 1 and gRNA target 2) targeting exon 2.

(B) Gel images of PCR products from individual larvae (two control larvae and three *cxcr4a* crispants) at 3 dpf for each gRNA target (upper: gRNA target 1, lower: gRNA target 2). Primer sites are shown in (A).

(C) PCR products in (B) were analyzed using a microchip electrophoresis system. The homoduplex peaks of PCR fragments (arrowheads) seen in controls were markedly attenuated in all *cxcr4a* crispant individuals. In contrast, heteroduplex signals (red arrows), which were not seen in controls, appeared in *cxcr4a* crispants (red arrows).

(D) Time-sequential images of a *Tg(kdrl:EGFP)* *cxcr4a* crispant larva (F0 injected larva) (3 dpf) after tip cell sprouting from the PHBC. Elapsed time (h:min). Similar to *cxcr4a* mutants, tip cells (arrowheads) do not migrate toward the anastomotic targets in *cxcr4a* crispants.

(E) Time-sequential images of high-speed 3D time-lapse imaging of a *Tg(fli1:Gal4FF);Tg(UAS:GCaMP7a);Tg(kdrl:TagBFP)* *cxcr4a* crispant larva (3 dpf) taken every 15 sec. Local Ca^2+^ oscillations are detected in protrusions of a tip cell (yellow arrowheads).

Scale bar:10 μm. LM, lower marker, UM, upper marker.

**Legends for Supplementary Table and Movies**

**Table S1 Sequences of oligonucleotides.**

Sequence list of CRISPR-Cas9 CrRNAs and primers.

**Movie 1 Non-directional and directional migration of a sprouting tip cell from the primordial hindbrain channels (PHBC) toward the central artery (CtA).**

Time-lapse recording in the hindbrain of a *Tg(kdrl:TagBFP);Tg(fli1:H2B-mCherry)* larva (from 3 dpf). Endothelial cells (ECs) and EC nuclei are labeled with TagBFP (cyan) and H2B-mCherry (magenta), respectively. Elapsed time (h:min). Dorsal view, anterior to the top.

**Movie 2 Non-directional migration of a sprouting tip cell from the PHBC in the *cxcr4a* mutant.**

Time-lapse recording in the hindbrain of a *Tg(kdrl:EGFP)* homozygous *cxcr4a* mutant (from 3 dpf). ECs are labeled with EGFP. Elapsed time (h:min). Dorsal view, anterior to the top.

**Movie 3 Ca^2+^ imaging of a sprouting tip cell in the non-directional phase.**

Time-lapse recording in the hindbrain of a *Tg(fli1:Gal4FF);Tg(UAS:GCaMP7a);Tg(kdrl:TagBFP)* larva during the non-directional phase (3 dpf). Elapsed time (sec). Local Ca^2+^ oscillations (yellow) are detected in the leading front of tip cell sprouting from the PHBC. Dorsal view, anterior to the top.

**Movie 4 Ca^2+^ imaging of a sprouting tip cell in the directional phase.**

Time-lapse recording in the hindbrain of a *Tg(fli1:Gal4FF);Tg(UAS:GCaMP7a);Tg(kdrl:TagBFP)* larva during the directional phase (3 dpf). Elapsed time (sec). Local Ca^2+^ oscillations (yellow) are not detected in the leading front of tip cell. Dorsal view, anterior to the top.
